# Association between Serum Bilirubin and Estimated Glomerular Filtration Rate among Elderly Persons

**DOI:** 10.1371/journal.pone.0115294

**Published:** 2014-12-16

**Authors:** Ryuichi Kawamoto, Daisuke Ninomiya, Yoichi Hasegawa, Yoshihisa Kasai, Tomo Kusunoki, Nobuyuki Ohtsuka, Teru Kumagi

**Affiliations:** 1 Department of Community Medicine, Ehime University Graduate School of Medicine, Ehime, Japan; 2 Department of Internal Medicine, Seiyo Municipal Nomura Hospital, Ehime, Japan; UNIFESP Federal University of São Paulo, Brazil

## Abstract

Chronic kidney disease (CKD) is a major public health problem. However, few studies have examined the significance of serum bilirubin as a risk factor for the development of CKD in the general Japanese population. The subjects comprised 413 men (mean age: 79±9 years; (range, 60–100 years) and 637 women (mean age: 81±8 years; range, 60–106 years) who visited the medical department of Seiyo Municipal Nomura Hospital. We examined the relationship between increased serum bilirubin and renal function that was evaluated by estimated glomerular filtration rate (eGFR) using CKD-EPI equations modified by a Japanese coefficient. Stepwise multiple regression analysis with eGFR as the objective variable, and adjusted risk factors as the explanatory variables, showed that serum bilirubin (*β* = 0.11, *P*<0.001) was significantly and independently associated with eGFR, in addition to gender, age, prevalence of antihypertensive medication, triglycerides, prevalence of antidiabetic medication, and serum uric acid. Compared with stages 1+2 (eGFR ≥60.0 ml/min/1.73 m^2^), mean multivariate-adjusted odds ratio {95% (confidence interval (CI)} for hypobilirubinemia (first quartile, **<**0.52 mg/dL) was 3.52 (range: 1.88–6.59). Next, to control potential confounding factors, data were further stratified by gender, age, medication (antihypertensive, antidyslipidemic, and antidiabetic agents), and prevalence of cardiovascular disease. The standardized coefficient for eGFR was significant in both groups, and there was no interaction between the groups. Our data demonstrated an independent positive association between serum bilirubin and eGFR in both genders. Low serum bilirubin level would be useful as a potential risk factor for renal function.

## Introduction

The increasing prevalence of chronic kidney disease (CKD) is a major public health problem [1]. There is now convincing evidence that CKD can be detected using simple laboratory tests and is associated with high annual rates of cardiovascular complications and all-cause mortality [2], [3], [4]. Identifying additional risk factors for CKD other than known risk factors such as reduced estimated glomerular filtration rate (eGFR) and increased urinary excretion of microalbumin may be helpful to predict future renal failure in CKD patients and prevent progression of the disease to renal failure.

Serum bilirubin, a major intravascular product of heme catabolism, has well-documented neurotoxic effects in infants [5], however, current evidences demonstrate that mildly elevated serum bilirubin is a potent antioxidant that may provide important protection against inflammation, cardiovascular disease (CVD), and all-cause mortality in adults [6], [7]. Several large studies have shown a positive relationship between serum bilirubin and eGFR [8. 9, 10,11], indicating that serum bilirubin has a potential renoprotective effect. However, Targher et al. found that serum bilirubin was negatively associated with eGFR, suggesting that serum bilirubin is a renal risk factor [12].

We have evaluated the relationship between serum bilirubin and potential risk factors such as hypertension, hyperglycemia, and lipids, as well as renal function, using cross-sectional data from elderly persons.

## Materials and Methods

### Subjects

Subjects for this investigation were recruited from among consecutive patients aged ≥60 years that visited the medical department of Seiyo Municipal Nomura Hospital. Participants with severe cardio-renal or nutritional disorders that would affect BP, lipid and glucose metabolisms were excluded. Thus, 1,050 elderly persons were enrolled in the study. All procedures were approved by the Ethics Committee of Seiyo Municipal Nomura Hospital, and written informed consent was obtained from each subject.

### Evaluation of Confounding Factors

Information on demographic characteristics and confounding factors was collected using the clinical files of the patients. Body mass index (BMI) was calculated by dividing weight (in kilograms) by the square of the height (in meters). We measured systolic blood pressure (SBP) and diastolic blood pressure (DBP) in the right upper arm of patients while in a sedentary position using a standard sphygmomanometer or an automatic oscillometric blood pressure recorder. Smoking status was defined as the number of cigarette packs per day multiplied by the number of years smoked (pack year) irrespective of the difference between current and past smoking status: never, light (<20 pack year), moderate (20–39 pack year), and heavy (≥40 pack year). Fasting total cholesterol (T-C), TG, high-density lipoprotein cholesterol (HDL-C), fasting plasma glucose (FPG), creatinine (enzymatic method), uric acid, and serum bilirubin were measured within 24 hours after admission. Low-density lipoprotein cholesterol (LDL-C) level was calculated by the Friedewald formula [13]. Patients with TG levels of ≥400 mg/dL were excluded. eGFR was calculated using CKD-EPI equations modified by a Japanese coefficient (eGFR_CKDEPI_): Male, Cr ≤0.9 mg/dl, 141× (Cr/0.9) ^−0.411^ ×0.993 ^age^ ×0.813; Cr>0.9 mg/dl, 141× (Cr/0.9) ^−1.209^ ×0.993 ^age^ ×0.813; Female, Cr ≤0.7 mg/dl, 144× (Cr/0.7) ^−0.329^ ×0.993 ^age^ ×0.813; Cr>0.7 mg/dl, 144× (Cr/0.7) ^−1.209^ ×0.993 ^age^ ×0.813 [14]. Histories of antihypertensive, antidyslipidemic, and antidiabetic medication use were also evaluated. Moreover, ischemic stroke, ischemic heart disease, and peripheral vascular disease were defined as CVD.

### Statistical analysis

All values are expressed as the mean ± standard deviation (SD), unless otherwise specified, and in the cases of parameters with non-normal distribution (such as TG, FPG, and serum bilirubin), the data are shown as median (interquartile range) values. In all the analyses, parameters with non-normal distributions were used after log-transformation. Statistical analysis was performed using IBM SPSS Statistics Version 21 (Statistical Package for Social Science Japan, Inc., Tokyo, Japan). Differences in means and prevalence among the groups were analyzed by Student's t-test for continuous data and χ^2^ test for categorical data, respectively. Pearson's correlations were calculated in order to characterize the associations between various characteristics and eGFR. Multiple linear regression analysis was used to evaluate the contribution of each confounding factor to eGFR. Subjects were divided into four groups based on stage of eGFR (stage 1, eGFR ≥90; stages 2, eGFR ≥60; stage 3a, 60.0 to 45.0; stage 3b, 44.9 to 30.0; stage 4, <30 mL/min/1.73 m^2^) and quartile of serum bilirubin (Quartile-1, 0.21–0.51; Quartile-2, 0.52–0.71; Quartile-3, 0.72–0.99; Quartile-4, 1.00–2.00 mg/dL), and logistic regression analyses were used to test significant determinants of CKD serving as the dichotomous outcome variable. To examine the consistency of the observed association between serum bilirubin levels and eGFR, we performed subgroup analyses by age (<80, ≥80 years), medication (such as antihypertensive, antidyslipidemic, and antidiabetic agents) (absent, present), and CVD (absent, present). Interaction between serum bilirubin and the subgroups was analyzed by a general linear model. A value of p<0.05 was considered significant.

## Results

### Subject background factors categorized by serum bilirubin level

Table 1 shows the value of each background factor categorized by serum bilirubin level. The subjects comprised 413 men aged 79±9 (range, 60–100) years and 637 women aged 81±8 (60–106) years. Mean eGFR in the study sample was 56.5 mL/min/1.73 m^2^ (SD, 18.8), with 1.0% in stage 1, 51.8% stage 2, 21.7% stage 3a, 14.7% stage 3b, and 10.9% stage 4. Though age, prevalence of antihypertensive medication, TG, serum uric acid, and serum creatinine level were significantly higher in the lower eGFR group, DBP, HDL-C, and LDL-C were significantly lower. There were no inter-group differences regarding gender, BMI, prevalence of smoking status, SBP, prevalence of antidyslipidemic medication, and FPG.

**Table 1:** Characteristics of various risk factors of the subjects by estimated glomerular filtration ratio.

CKD group (mL/min/1.73 m^2^)	Stages 1+2	Stage 3a	Stage 3b	Stage 4	
eGFR by CKD-EPI equation	≥60.0	59.9–45.0	44.9–30.0	30.0>	*P* for trend*
Characteristic N = 1,050	N = 554	N = 228	N = 154	N = 114	
Gender male, (%)	41.0	41.2	36.4	31.6	0.220
Age (years)	79±8	82±8	85±7	84±8	<0.001
Body mass index† (kg/m^2^)	21.1±3.7	21.9±4.0	21.6±4.1	21.6±4.4	0.060
Smoking status‡ (%)	75.1/3.6/9.2/12.1	80.7/2.2/7.0/10.1	78.6/1.3/7.8/12.3	80.7/2.6/5.3/11.4	0.676
Systolic blood pressure (mmHg)	137±25	138±24	134±25	131±31	0.087
Diastolic blood pressure (mmHg)	76±14	77±14	73±15	69±14	<0.001
Antihypertensive medication (%)	48.0	57.0	68.2	67.5	<0.001
Triglycerides (mg/dL)	73 (57–102)	81 (62–109)	82 (64–116)	101 (68–139)	<0.001
HDL cholesterol (mg/dL)	56±17	56±17	53±18	51±19	0.013
LDL cholesterol (mg/dL)	106±38	105±34	99±36	98±36	0.048
Antidyslipidemic medication (%)	7.9	10.1	5.2	9.6	0.348
Fasting blood glucose (mg/dL)	115 (99–147)	119 (98–145)	122 (100–157)	126 (104–148)	0.217
Antidiabetic medication (%)	19.9	17.1	18.2	31.6	0.012
Serum uric acid (mg/dL)	4.6±1.5	5.5±1.5	6.6±1.9	8.0±2.5	<0.001
Serum creatinine (mg/dL)	0.67±0.14	0.93±0.14	1.20±0.20	2.47±1.34	<0.001
Cardiovascular disease (%)	38.3	45.2	52.6	56.1	<0.001

Data are means ± standard deviation. HDL, high-density lipoprotein; LDL, low-density lipoprotein; eGFR, estimated glomerular filtration rate. †Body mass index was calculated using weight in kilograms divided by the square of the height in meters. ‡Smoking status: daily consumption (pack) × duration of smoking (year) {never, light (<20 pack year), moderate (20–39 pack year), heavy (≥40 pack year)}. Data for triglycerides, fasting plasma glucose, and serum bilirubin were skewed and are presented as median (interquartile range) values which were then log-transformed for analysis. *1-way ANOVA test was used for the continuous data and χ^2^ test for the categorical data.

### Estimated GFR of subjects categorized by serum bilirubin level

Serum bilirubin levels decreased progressively with decreasing eGFR (Table 2), especially for the stage 3b or 4 group versus the stages 1+2 group, and the stage 4 versus the stages 1+2 to 3a groups. Moreover, the higher stage 4 group had a higher prevalence of participants with a serum bilirubin of <0.52 mg/dL.

**Table 2:** Serum bilirubin and prevalence of hypobilirubinemia in the subjects by estimated glomerular filtration ratio.

CKD stage (mL/min/1.73 m^2^)	Stages 1+2	Stage 3a	Stage 3b	Stage 4	
eGFR by CKD-EPI equation	≥60.0	59.9–45.0	44.9–30.0	30.0>	*P* for trend*
Characteristic N = 1,050	N = 554	N = 228	N = 154	N = 114	
Serum bilirubin (mg/dL)	0.73 (0.55–1.00)	0.70 (0.52–0.98)	0.64 (0.46–0.93) ^a^	0.57 (0.42–0.77) ^b, c^	<0.001
Hypobilirubinemia (%)	21.1	22.8	29.2	46.5	<0.001
(First quartile, <0.52 mg/dL)					

Data for serum bilirubin was skewed and presented as median (interquartile range) values, which were then log-transformed for analysis. *1-way ANOVA test was used for the continuous data and χ^2^ test for the categorical data. ^a^: *P*<0.05; ^b^: *P*<0.001 versus Stages 1+2. ^c^: *P*<0.001 versus Stage 3a.

### Relationship of risk factors, including serum bilirubin and eGFR

**Figure 1:**
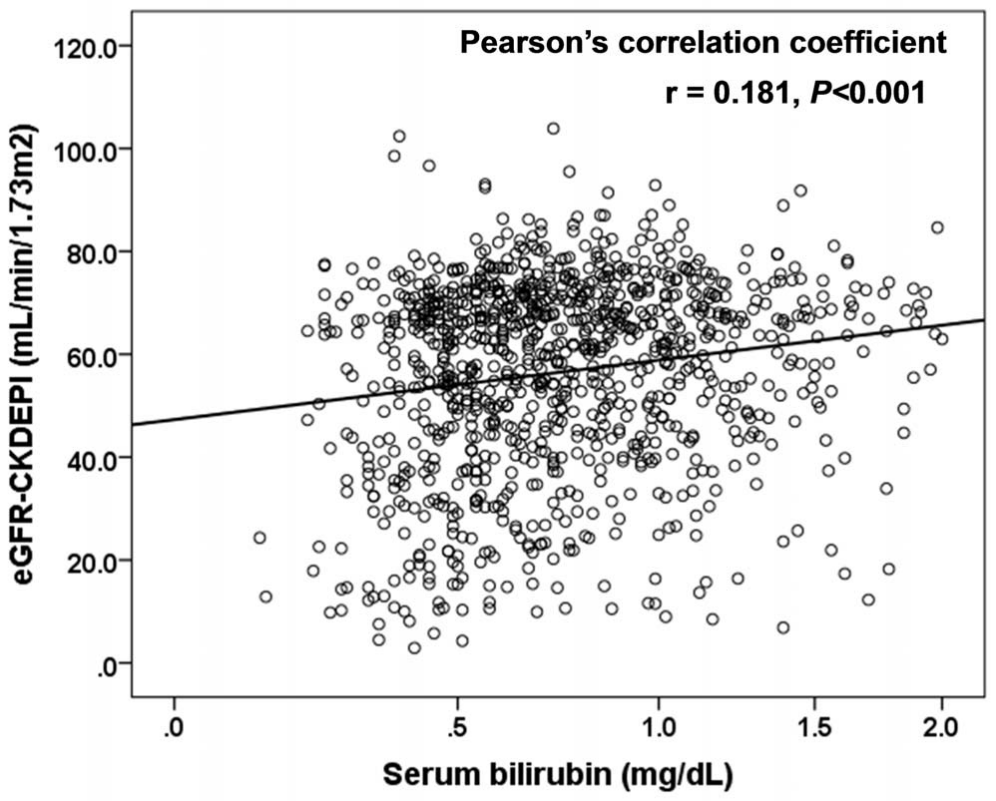
Relationship between serum bilirubin and estimated glomerular filtration rate (eGFR). eGFR was calculated using CKD-EPI equations modified by a Japanese coefficient (eGFR_CKDEPI_): Male, Cr ≤0.9 mg/dl, 141× (Cr/0.9) ^−0.411^ ×0.993 ^age^ ×0.813; Cr>0.9 mg/dl, 141× (Cr/0.9) ^−1.209^ ×0.993 ^age^ ×0.813; Female, Cr ≤0.7 mg/dl, 144× (Cr/0.7) ^−0.329^ ×0.993 ^age^ ×0.813; Cr>0.7 mg/dl, 144× (Cr/0.7) ^−1.209^ ×0.993 ^age^ ×0.813. Serum bilirubin was skewed and log-transformed for analysis.

Table 3 shows the relationship between participant characteristics and eGFR. Bilirubin (r = 0.18, *P*<0.001) along with gender, age, BMI, DBP, prevalence of antihypertensive medication, TG, HDL-C, and serum uric acid correlated significantly with eGFR (Fig. 1). Stepwise multiple regression analysis using eGFR as an objective variable, and bilirubin, gender, age, prevalence of antihypertensive medication, TG, prevalence of antidiabetic medication, and serum uric acid adjusted for risk factors as explanatory variables, showed that serum bilirubin (β = 0.11, P<0.001) was significantly and independently associated with eGFR, in addition to gender, age, prevalence of antihypertensive medication, TG, prevalence of antidiabetic medication, and serum uric acid.

**Table 3:** Relationship between various risk factors including serum bilirubin and estimated glomerular filtration rate.

		Multiple linear regression	
	Pearson's correlation	Forced method	Stepwise method
Characteristic	r (*P*-value)	*β* (*P*-value)	*β* (*P*-value)
Gender (male = 0, female = 1)	−0.08 (0.007)	−0.10 (<0.001)	−0.08 (<0.001)
Age	−0.36 (<0.001)	−0.29 (<0.001)	−0.29 (<0.001)
Body mass index	−0.06 (0.039)	−0.04 (0.123)	------
Smoking status	0.05 (0.108)	−0.03 (0.272)	------
Systolic blood pressure	0.05 (0.137)	------	------
Diastolic blood pressure	0.17 (<0.001)	0.04 (0.101)	------
antihypertensive medication	−0.18 (<0.001)	−0.07 (0.002)	−0.08 (<0.001)
Triglycerides	−0.18 (<0.001)	−0.06 (0.021)	−0.06 (0.010)
HDL cholesterol	0.10 (0.001)	0.00 (0.869)	------
LDL cholesterol	0.05 (0.110)	0.03 (0.327)	------
Antidyslipidemic medication	0.00 (0.921)	−0.02 (0.434)	------
Fasting blood glucose	−0.04 (0.171)	−0.02 (0.471)	------
Antidiabetic medication	−0.06 (0.058)	−0.07 (0.010)	−0.08 (0.001)
Serum uric acid	−0.58 (<0.001)	−0.52 (<0.001)	−0.53 (<0.001)
Serum bilirubin	0.18 (<0.001)	0.11 (<0.001)	0.11 (<0.001)
R^2^	------	0.47 (<0.001)	0.47 (<0.001)

r, Pearson's correlation coefficient; β, standardized coefficient; R^2^, multiple coefficient of determination. Data for triglycerides, fasting plasma glucose, and serum bilirubin were skewed and log-transformed for analysis.

### Association between serum bilirubin categories and risk for stage 3 or stage 4 reduced eGFR

Table 4 shows the odds ratio of renal dysfunction for each quartile increase in serum bilirubin and presence of hypobilirubinemia. The non-adjusted, age and gender-adjusted, and multivariate-adjusted odds ratios {95% confidence interval (CI)} of stage 4 for each quartile in serum bilirubin were 0.62 (0.51−0.76), 0.66 (0.53−0.80), and 0.62 (0.46−0.83), respectively. Moreover, compared with stages 1+2, the non-adjusted, age and gender-adjusted, and multivariate-adjusted odds ratios (95% CI) of stage 4 for hypobilirubinemia were 3.25 (2.13−4.94), 2.97 (1.91−4.61), and 3.52 (1.88−6.59), respectively.

**Table 4:** Odds ratio of renal dysfunction for decrease in serum bilirubin and hypobilirubinemia.

	Odds ratio (95% confidence interval)			
CKD stage (mL/min/1.73 m^2^)	Stages 1+2	Stage 3a	Stage 3b	Stage 4
eGFR by CKD-EPI equation	≥60.0	59.9−45.0	44.9−30.0	<30.0
Characteristic N = 1,050	N = 554	N = 228	N = 154	N = 114
Serum bilirubin (per quartile)	1.00 (referent)	0.96 (0.83−1.11)	0.82 (0.69−0.97)	0.62 (0.51−0.76)
Non-adjusted	1.00 (referent)	0.98 (0.84−1.13)	0.86 (0.73−1.03)	0.66 (0.53−0.80)
Gender & age-adjusted	1.00 (referent)	0.96 (0.82−1.12)	0.93 (0.75−1.15)	0.62 (0.46−0.83)
Multivariate-adjusted*				
Hypobilirubinemia	1.00 (referent)	1.10 (0.76−1.60)	1.54 (1.03−2.31)	3.25 (2.13−4.94)
(First quartile, **<**0.52 mg/dL)	1.00 (referent)	1.07 (0.74−1.57)	1.29 (0.84−1.98)	2.97 (1.91−4.61)
Non-adjusted	1.00 (referent)	1.12 (0.75−1.68)	1.03 (0.61−1.74)	3.52 (1.88−6.59)
Age & gender-adjusted				
Multivariate-adjusted*				

*Models adjusted for gender, age, body mass index, smoking status, diastolic blood pressure, antihypertensive medication, triglycerides, high density lipoprotein cholesterol, low density lipoprotein cholesterol, antidyslipidemic medication, fasting blood glucose, antidiabetic medication, and uric acid. Data for triglycerides, and fasting plasma glucose were skewed and log-transformed for analysis. Serum bilirubin: quartile-1, 0.21−0.51; quartile-2, 0.52−0.71; quartile-3, 0.72−0.99; quartile-4, 1.00−2.00 mg/dL.

### Relationship between serum bilirubin and eGFR within selected subgroups

Next, to control potential confounding factors, the data were further stratified by gender, age, medication (antihypertensive, antidyslipidemic, and antidiabetic agents), and prevalence of CVD (Table 5). The standardized coefficient for eGFR was significant in all of two groups, and there was no interaction between two groups.

**Table 5:** Relationship between serum bilirubin and estimated glomerular filtration rate within selected subgroups.

Characteristics	N	*β* (*P*-value)	*P-value*	*P*-interaction
Gender				
Men	413	0.11	0.008	0.778
Women	637	0.10	0.001	
Age				
<80 years	415	0.13	0.002	0.605
≥80 years	635	0.10	0.001	
Medication				
Absence	390	0.15	<0.001	0.463
Presence	660	0.08	0.005	
Cardiovascular disease				
Absence	590	0.07	0.034	0.080
Presence	460	0.14	<0.001	

β, standardized coefficient. Medication included antihypertensive, antidyslipidemic, and antidiabetic medications. §Adjusted for all confounding factors in Table 3 by multiple linear regression analysis (Forced method).

## Discussion

To examine any possible contribution of decreased serum bilirubin to decreased renal function among elderly persons, we studied the relationship between risk factors including serum bilirubin and eGFR. This study showed a graded decrease in eGFR with decreasing serum bilirubin values starting at 0.52 mg/dL. Individuals with hypobilirubinemia (first quartile of serum bilirubin, **<**0.52 mg/dL) were at a higher risk of stage 4 CKD. Surprisingly, multiple linear regression analysis revealed that the strength of serum bilirubin level as an independent determinant of eGFR was similar to those of known factors such as gender, age, prevalence of antihypertensive medication, TG, prevalence of antidiabetic medication, and serum uric acid. To our knowledge, few epidemiologic studies have quantified the link between decreased serum bilirubin and renal function in population-based settings in Japan.

Several studies have shown that decreased serum bilirubin is positively associated with the incidence of CKD. In a community-based cross-sectional study in Korean non-diabetic and diabetic adults (mean age: 55.6±14.1 years; 49.1% male) in Korea, Shin et al [9] found that total serum bilirubin level was negatively correlated with 24-hour urine protein and positively correlated with eGFR after adjustment for potential confounding factors. Fukui et al. [8] found that serum bilirubin was independently and negatively associated with albuminuria in a hospital-based sample of 633 Japanese type 2 diabetic patients (mean age: 64.4±11.5 years; 52% male). In a Japanese cohort study performed on a consecutive series of 2,784 subjects without CKD (58.4% male) at baseline, serum bilirubin level was suggested as a novel risk factor for the progression of CKD, defined as eGFR <60 ml/min/1.73 m^2^ [10]. A longitudinal cohort study of 12,823 Korean male workers aged 30 to 59 years without CKD or proteinuria at baseline reported that higher serum direct bilirubin levels were significantly associated with a lower risk of developing CKD defined as eGFR <60 ml/min/1.73 m^2^, even following adjustments for important confounders [11]. However, neither serum total nor indirect bilirubin level was significantly associated with incidence of CKD. The findings of Targher et al. [12], however, are in contrast to these. In their observational hospital-based sample of 2,678 US adult outpatients (mean age: 55±18 years; 43% male) they found that serum bilirubin was inversely associated with eGFR in both non-diabetic (r = **−**0.17; *p*<0.0001) and diabetic patients (r = -0.14; *p*<0.05). However, in that study, no adjustment was made for important confounders. In our hospital-based sample of 1,050 elderly persons, we found that multivariate adjusted-odds ratio of hypobilirubinemia for stage 4 (eGFR <30 ml/min/1.73 m^2^) was 3.52 (1.88−6.59).

Based on in vitro as well as animal studies, serum bilirubin is generally recognized as an antioxidant substance [15]. In 70 otherwise healthy full-term newborns with neonatal hyperbilirubinemia and 20 control newborns without jaundice, plasma bilirubin showed significant negative correlation with malondialdehyde but positive correlation with antioxidant enzyme activities (such as superoxide dismutase, catalase and glutathione peroxidase levels) [16]. Several studies have demonstrated that serum bilirubin is also negatively associated with CVD [17], [18], [19], [20]. We [21] also reported that high serum bilirubin level is negatively associated with carotid atherosclerosis among elderly persons as well as the population of our study. These results demonstrate a protective effect of serum bilirubin on atherosclerosis. Balloon injury-induced neointima formation was less in genetically hyperbilirubinemic Gunn rats and wild-type rats treated with biliverdin, the precursor of bilirubin, than in controls, and both bilirubin and biliverdin inhibited vascular smooth muscle cell proliferation [22]. Moderate hyperbilirubinemia decreases blood pressure in ANG II-dependent hypertension through mechanisms that decrease oxidative stress [23], and improvement of renal hemodynamics may be one mechanism by which moderate hyperbilirubinemia lowers blood pressure in this model [24]. Moreover, serum unconjugated bilirubin inhibits TNFalpha-related induction of endothelial adhesion molecules and has a protective effect against the development of atherosclerosis [25]. We were unable to assess the antioxidant effect of serum bilirubin, as tests for oxidative stress markers and antioxidant enzymes were not carried out. The results of our study, however, suggest that hypobilirubinemia, which may indicate increased oxidative stress, may be a possible risk factor of CKD independent of other confounding factors.

Some limitations of this study must be considered. First, the cross-sectional study design is limited in the ability to eliminate causal relationships between serum bilirubin and eGFR. Second, estimating GFR tends to be less accurate in subjects with normal renal function and CKD than GFR when inulin clearance is used, but is more accurate than serum creatinine or the Cockcroft-Gault equation [26]. Third, our definition of eGFR is based on a single assessment of serum creatinine, which may introduce a misclassification bias. Fourth, in this study, CKD may have been misclassified with eGFR>60 ml/min/1.73 m^2^ and proteinuria as mildly reduced renal function because reduced renal function was defined as reduced eGFR irrespective of the presence or absence of proteinuria. Therefore, the demographics and referral source may limit generalizability.

## Conclusions

In conclusion, the present study showed that decreased serum bilirubin level is strongly associated with decreased eGFR in the elderly. The underlying mechanism behind this relationship is unclear, but seems to be independent of traditional cardiovascular risk factors such as gender, age, hypertension, dyslipidemia, and diabetes. For community-dwelling healthy persons, prospective population-based studies are needed to investigate the mechanisms underlying this association.
